# Supplementary material for: A non-canonical striatopallidal Go pathway that supports motor control
Source: Nat Commun. 2023 Oct 23;14:6712. doi: 10.1038/s41467-023-42288-1 (PMC10593790; doi:10.1038/s41467-023-42288-1)
Supplement: Supplementary file 1 — Supplementary Information [file 41467_2023_42288_MOESM1_ESM.pdf]

Cohort	N
Drd1-cre, GPe/SNr retrograde tracing	3
Drd1-cre, GCaMP7s	7 rotarod, 8 open field
Drd1-cre, SyGCaMP6s	5
Drd1-cre, SyGCaMP8s	7
C57Bl6/J wild-type, [3H]-CNO	3
Drd1-cre, hM4D-mCherry	9 rotarod, 10 open field
Drd1-cre, mCherry	9 rotarod, 7 open field
Drd1-cre, ChR2 + hM4D	5 Sal GPe, 5 CNO GPe, 5 Sal SNr, 5 CNO SNr
Drd1-cre, eOPN3-mScarlet<br>Drd1-cre, GFP	10 rotarod, 8 open field<br>9 rotarod, 9 open field
Drd1-cre;Npas1-cre, ChrimsonR;GCaMP6s<br>Drd1-cre;Npas1-cre, mCherry;GCaMP6s	5<br>6
Npas1-cre, YFP<br>Npas1-cre, ChR2-YFP	12 open field, 8 rotarod<br>13 open field, 9 rotarod
ChAT-cre, YFP<br>ChAT-cre, ChR2-YFP	8<br>8

Coordinates AAV	Site A	Site B	Volume/Site
DMS AAV5 or AAV9 (2 sites/hemis.)	AP 0.8; ML +/-1.8; DV 3.4	AP 1.1; ML +/-1.4; DV 3.4	200 nL
DMS AAV1 (4 sites/hemis.)	AP 0.7 or 0.9; ML +/-1.9; DV 3.1	AP 0.7 or 0.9; ML +/-1.5; DV 3.1	150 nL
DMS+DLS AAV5 (2 sites/hemis.)	AP 0.8; ML +/-2.0; DV 3.2	AP 1.1; ML +/-1.4; DV 3.2	400 nL
GPe Npas1-cre AAV5 or Drd1-cre rgHSV	AP -0.45; ML +/-1.9; DV 3.75	.	150 nL
GPe ChAT-cre AAV5	AP -1.0; ML +/-2.3; DV 3.7	.	100 nL
SNr retrobeads	AP -3.15; ML +/-1.3; DV 4.65	.	75 nL

Coordinates implants	Site
DMS optic fibers	AP +0.9; ML +2.6, DV -3.1 at 15 degree angle
GPe fluid cannulas	AP -0.45; ML +/-2.0; DV 2.1 (+1.5mm removable inj. cannula)
GPe optic fibers or electrodes	AP -0.45; ML +/-1.9; DV 3.75
SNr optic fibers or electrodes	AP -3.15; ML +/-1.35; DV 4.65

Supplementary Table S1. Summary table giving animal numbers and injection/implant coordinates for each cohort (note: the full references and titers for viruses are reported in Supplementary Dataset 1 as a separate file)

Virus name, and titer used	Reference	Identifier or Ref. Number and titer of the stock
rgHSV-EF1a-LS1L-eNpHR3.0-YFP (LS1L = cre-dependent)<br>(diluted 1:1 AAV:PBS)	Dr. Rachael Neve, Viral Gene Transfer Core, Mass Gen Hospital<br>(Neve and Lim, 1999)	Cat#RN417<br>Titer 5 x10^9 vg/mL
AAV9-Syn-DIO-jGCaMP7s<br>(diluted 1:1 AAV:PBS)	(Dana et al., 2019)	Addgene<br>Cat#104491-AAV9 (RRID Addgene_104491)<br>Lot# v34775<br>Titer 3.00 x10^13 vg/mL
AAV5-Syn-DIO-Synaptophysin-GCaMP6s<br>(used undiluted)	Plasmid provided by Ofer Yizhar. AAV made at Virovek<br>(Mahn et al., 2016)	Virovek<br>Lot# 19-141<br>Titer 2.04 x10^13 vg/mL
AAV9-CAG-DIO-Synaptophysin-jGCaMP8s<br>(used undiluted)	This paper	ETH Zurich Vector and Virus Production Platform (VVPP)<br>Lot# 037<br>Titer 2.5 x10^12 GC/mL
AAV5-Syn-DIO-GCaMP6s<br>(used undiluted)	(Chen et al., 2013)	Addgene<br>Cat#100845-AAV5 (RRID Addgene_100845)<br>Lot# v19064<br>Titer 1.10 x10^13 vg/mL
AAV5-hSyn-DIO-mCherry<br>(used undiluted)		Addgene<br>Cat#50459-AAV5 (RRID Addgene_50459)<br>Lot# v7465<br>Titer 4.8 x10^12 vg/mL<br>Lot# v120251<br>Titer 2.4 x10^13 vg/mL
AAV5-hSyn-DIO-hM4D<br>(used undiluted)<br>(diluted 1:1 AAV-DIO-hM4D:AAV-DIO-ChR2)	(Armbruster et al., 2007)	Addgene<br>Cat#43362-AAV5 (RRID Addgene_44362)<br>Lot# v4778<br>Titer 4.7 x10^12 vg/mL<br>Lot#v120251<br>Titer 2.4 x10^13 g/mL
AAV1-hSyn-DIO-EGFP<br>(used undiluted)		Addgene<br>Cat#50457-AAV5 (RRID Addgene_50457)
AAV1-hSyn1-SIO-eOPN3-mScarlet<br>(diluted 1:10 AAV:PBS)	AAV provided by Ofer Yizhar<br>(Mahn et al., 2021)	(Note: same construct now available at Addgene #125713, RRID Addgene_125713)
AAV5-Syn-DIO-ChrimsonR-tdTomato<br>(used undiluted)	(Klapoetke et al., 2014)	Addgene<br>Cat#62723-AAV5 (RRID Addgene_62723)<br>Lot# v63071<br>Titer 8.5 x10^12 vg/mL
AAV5-Ef1a-DIO-EYFP<br>(used undiluted)	UNC Vector Core	RRID Addgene_27056<br>Lot# AV4310L<br>Titer 4 x10^12 vg/mL
AAV5-EF1a-DIO-ChR2<br>(used undiluted)<br>(diluted 1:1 AAV-DIO-hM4D:AAV-DIO-ChR2)	UNC Vector Core<br>(Boyden et al., 2005)	RRID Addgene_20298<br>Lot# AV4313Z<br>Titer 3.2 x10^12 vg/mL<br>Lot# AV4313Y<br>Titer 4.8 x10^12 vg/mL<br>Lot# AV4313-2A<br>Titer 4 x10^12 vg/mL

Supplementary Table S2. Summary table giving references and titers for viruses used.

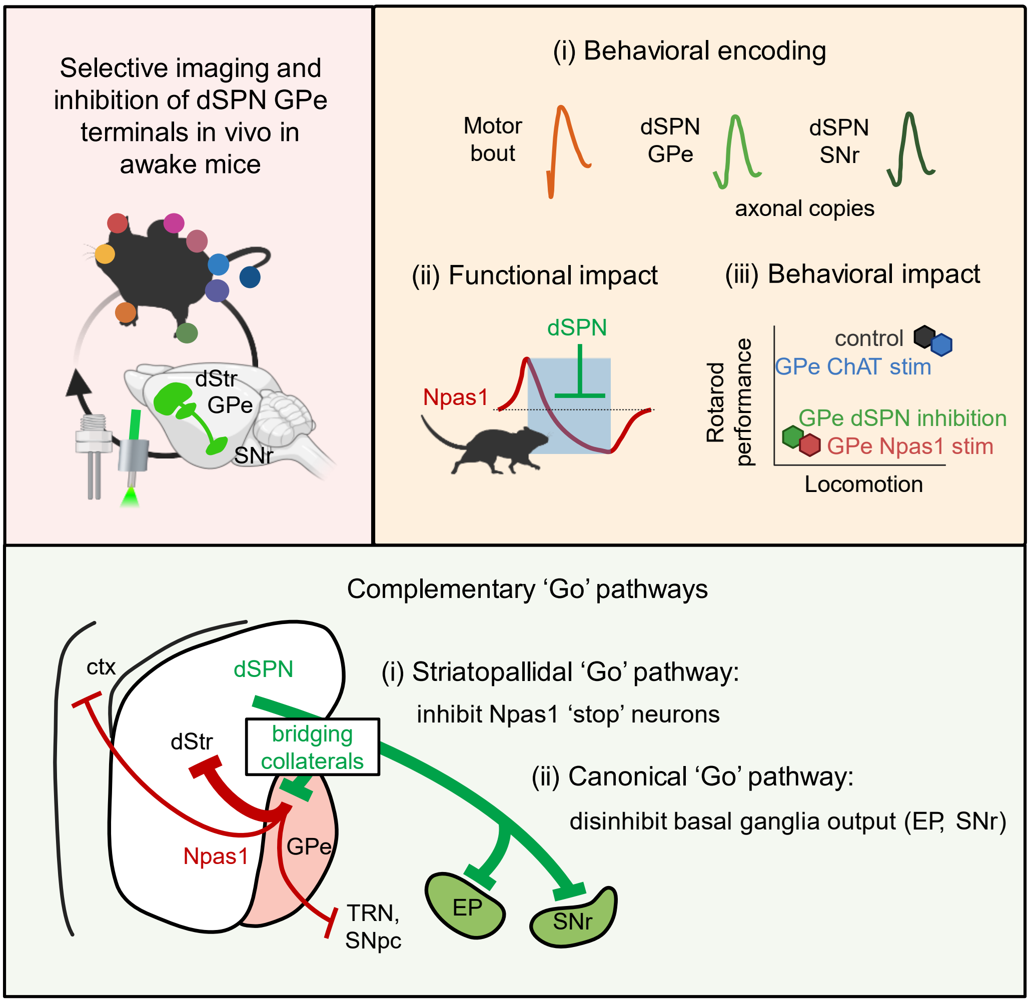

Supplementary Fig. S1. Summary of findings in graphical form. In the classical model of the basal ganglia direct and indirect pathways are functionally opposing based on their segregated connectivity to the substantia nigra reticulata, (SNr) versus globus pallidus externus (GPe). Here, we show that direct pathway striatal projection neurons (dSPNs) send synchronous motor-related activity to the GPe via axonal collaterals, inhibiting a Npas1 stop circuit (Npas1+ cells) to support motor function. We term this dSPN→GPe pathway the striatopallidal Go pathway. (Of note: Npas1 neurons project to the dorsal striatum dStr and this projection comprises arkypallidal neurons which send a stop signal to the dStr, but Npas1 neurons also project to the cortex ctx, thalamic reticular nucleus TRN and substantia nigra pars compacta SNpc, of which the functional relevance is still unclear). ChAT: choline acetyltransferase, labeling ChAT+ cholinergic interneurons. EP: entopeduncular nucleus.

A Injections of GCAMP AAVs primarily target the dorsomedial striatum (DMS)

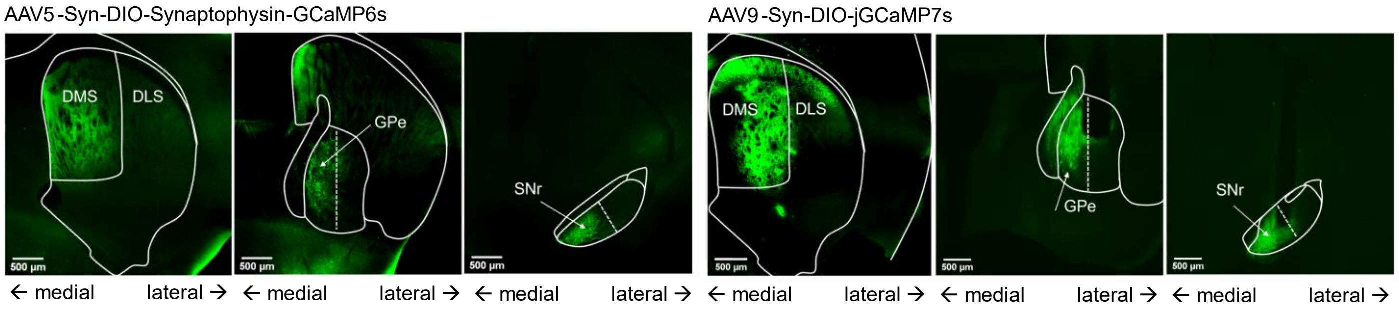

Supplementary Fig. S2. Anatomical evidence for bridging collaterals arising from dSPNs in the dorsomedial striatum (DMS) (Related to Fig. 2, 3 and Supplementary Fig. S4,S5). A. AAV injections of calcium indicators GCaMP primarily target the dorsomedial part (DMS) of the dorsal striatum (dStr). Cre-dependent (flexed) adenoassociated viruses (AAVs) expressing Synaptophysin-GCaMP6s (AAV5-Syn-DIO-Synaptophysin-GCaMP6s) (N=5 mice) or jCGaMP7s (AAV9-Syn-DIO-jGCaMP7s) (N=7 mice) were injected into the DMS. GFP expression largely avoids the dorsolateral striatum (DLS). Given the topographical organization of direct pathway striatal projection neuron (dSPN) projections in the globus pallidus externus (GPe) and substantia nigra reticulata (SNr) subregions [16], this leads to axonal projections primarily in the medial GPe and medial SNr, rather than their lateral parts.

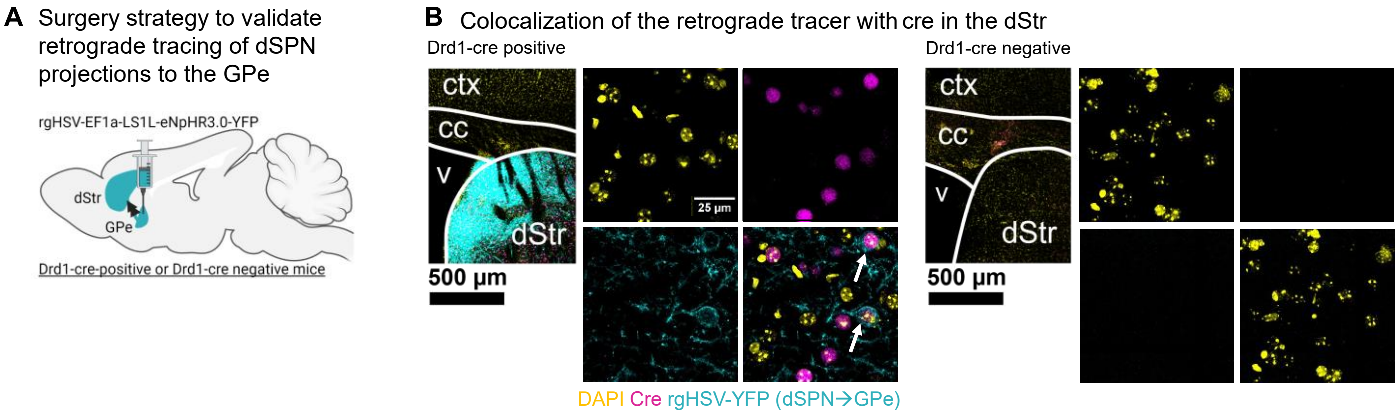

Supplementary Fig. S3. Validation of retrograde tracing strategy to trace dSPN projections from dStr to GPe. (Related to Fig. 1). A. Surgery strategy showing bilateral injection of the cre-dependent retrograde tracer rgHSV-EF1a-LS1L-eNpHR3.0-YFP into the GPe of Drd1-cre positive and negative mice. This labels neurons projecting to the globus pallidus externus (GPe) traced back to the dorsal striatum (dStr) where it should recombine only in Drd1 cells expressing cre in Drd1-cre-positive mice (not in Drd1-cre-negative mice). **B.** Left: Representative images of the dorsal striatum (dStr) showing YFP fluorescence (cyan) and cre expression (pink) in Drd1-cre positive mice. Zoom-in images show colocalization of YFP (cyan) with cre (pink) in Drd1-cre positive mice (white arrows) (left). No YFP+, cre- cells were detected in Drd1-cre positive mice. Right: same shown in Drd1-cre negative mice. No fluorescence was detected in Drd1-cre negative mice (representative images out of 6 sections examined). This indicates lack of recombination in cre-negative mice/Drd1-cre negative cells. Zoom-in images show no YFP or cre fluorescence detected in Drd1-cre negative mice (right). This indicates that the retrograde tracer only recombines in cre-positive cells. Images from N=1 Drd1-cre positive and N=1 Drd1-cre negative mice. Yellow: DAPI. ctx: cortex, cc: corpus callosum, dSPN: direct pathway striatal projection neurons, rgHSV: retrograde herpes simplex virus, v: ventricle.

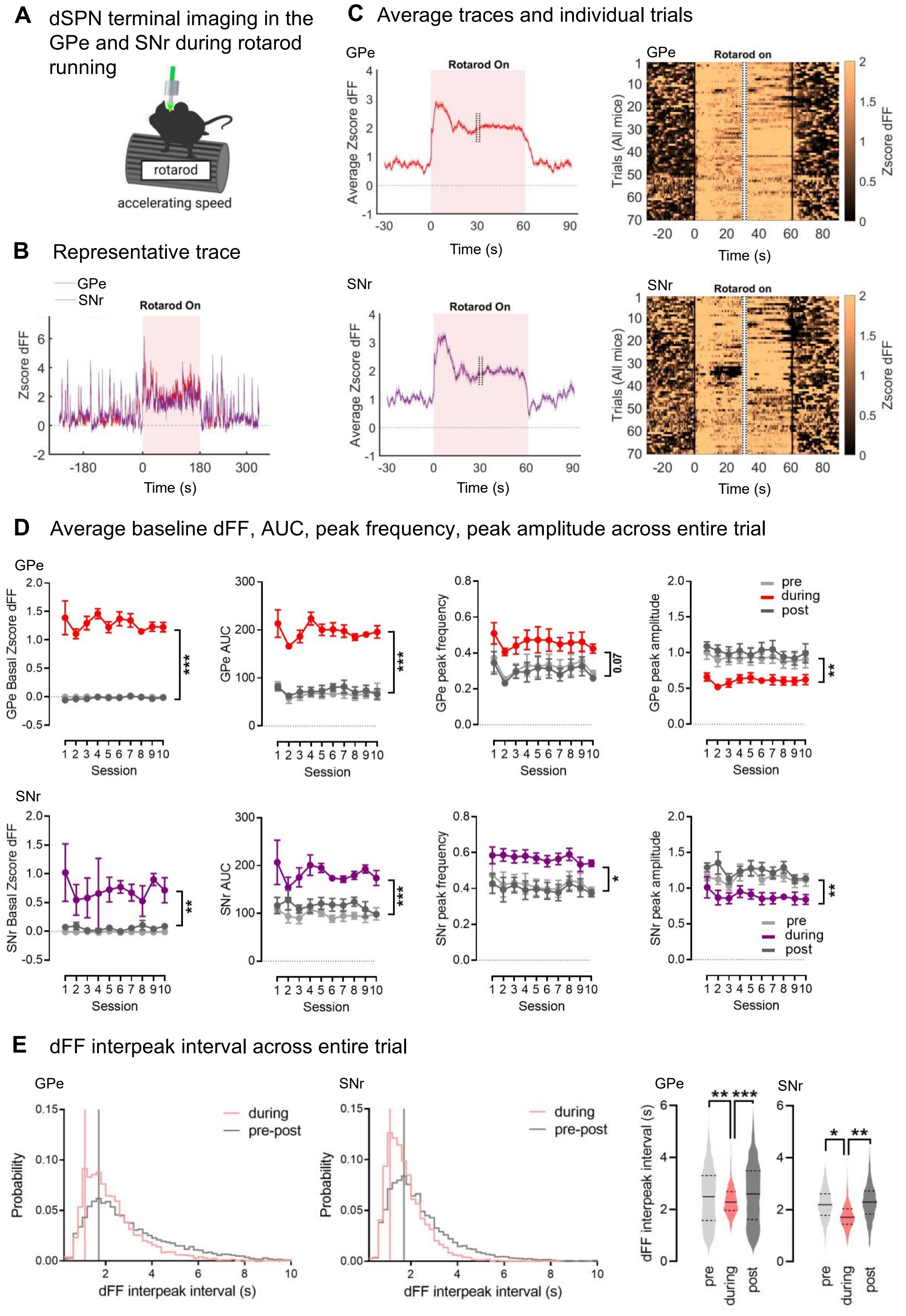

Supplementary Fig. S4. dSPN GPe and SNr axons show sustained activity during rotarod running (Related to Fig. 3). A. Mice for dual globus pallidus externus (GPe) / substantia nigra reticulata (SNr) direct pathway striatal projection neuron (dSPN) axonal imaging (using the calcium indicator jGCaMP7s) are video-recorded in the rotarod set at accelerating speed. **B.** Representative trace of GPe and SNr normalized fluorescence i.e. deltaF/F (dFF) in the rotarod task. **C.** Average GPe/SNr dFF traces (left) and heatmaps of all individual trials (10/animal) (right). Only the first and last 30 sec of the rotarod epoch is shown (indicated by the dashed lines) **D.** Basic properties of GPe and SNr Zscore dFF across the 10 trials, show a significant increase in baseline (Mixed ANOVA: epoch: GPe or SNr $p<0.001$; Sidak post-hoc pre/post vs during: GPe ***$p<0.001$, SNr **$p<0.01$), area under the curve (AUC) (Mixed ANOVA: epoch: GPe

or SNr p<0.001; Sidak post-hocs pre/post vs during: GPe and SNr ***p<0.001) and frequency (Mixed ANOVA: epoch: GPe p=0.0744, SNr p<0.01; Sidak post-hocs pre/post vs during: SNr *p<0.05) and a decrease in peak amplitude (Mixed ANOVA: epoch: GPe p<0.001, SNr p<0.01; Sidak post-hocs pre/post vs during: GPe and SNr **p<0.01) in the run epoch (during) vs rest (pre/post). For peak frequency and amplitude calculation, dFF was low-pass filtered using a 0.5s moving average smoothing to avoid overcounting peaks not relevant to the behavior of interest (jumps). **E.** Left: Probability distributions of dFF interpeak intervals (IPI, shown in 3B.) are computed by pooling IPIs across pre/post or during (run) epochs (all animals). The maximal probability (vertical bar) is reached at smaller IPIs in the running epoch vs pre/post epoch. Right: Average IPI (averaged per animal) significantly decreases during running (ANOVA: epoch: GPe p<0.001, SNr p<0.01; Sidak post-hocs: *p<0.05, **<0.01, ***<0.001). For peak frequency and amplitude calculation, dFF was low-pass filtered using a 0.5s moving average smoothing. N= 7 mice throughout. Data is mean±SEM. Exact p-values are given in **Supplementary Dataset S2.** Source data are provided as a Source Data file.

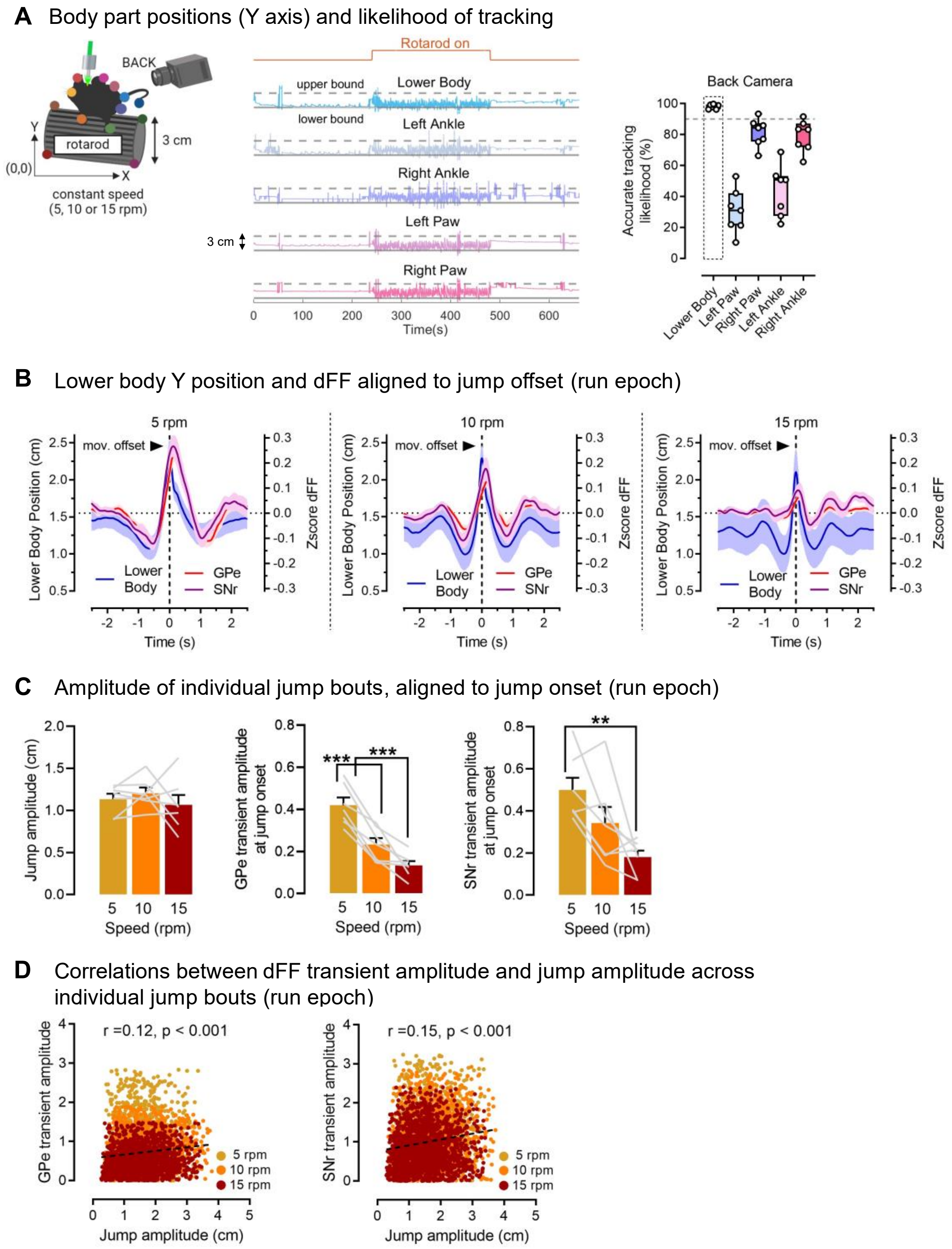

Supplementary Fig. S5. dSPN GPe and SNr axons track the temporal boundaries of individual motor bouts (Related to Fig. 3). A. Left: Representative trace of body part positions on the Y axis across 1 entire trial. Dashed lines show the upper and lower bounds of the rotarod (3 cm). Right: Average likelihood across entire trials shows higher tracking quality for the lower body (back camera). Note: Position changes across the Y axis could also be observed for the feet (like for the lower body, see main text), but due to suboptimal feet tracking (low likelihood), we focused our analyses on the lower body, rather than the feet to track jumping behavior. **B.** Average calcium recording (using the calcium indicator jGCaMP7s and behavioral data aligned to the offset of individual jump bouts (blue) during running in constant speed trials. Globus pallidus externus (GPe, red) and substantia nigra reticulata (SNr, purple) normalized fluorescence i.e. deltaF/F (dFF) decreases at jump offset, showing shorter and smaller transients with increasing rotarod speed. Data is mean±SEM. **C.** Amplitude of GPe/SNr dFF transients significantly decrease with rotarod speed (all: ANOVA: speed: $p<0.01$, Tukey post-hocs: *$p<0.05$, **<0.01, ***<0.001). Amplitude of jumps is unchanged (ANOVA: speed: $p=0.54$) **D.** Amplitude of individual jumps significantly correlate with amplitude of individual GPe/SNr dFF transients (Pearson r, all $p<0.001$: GPe: $r=0.12$; SNr: $r=0.15$). N=7 mice. Data is mean±SEM. Exact p-values are given in **Supplementary Dataset S2.** Source data are provided as a Source Data file.

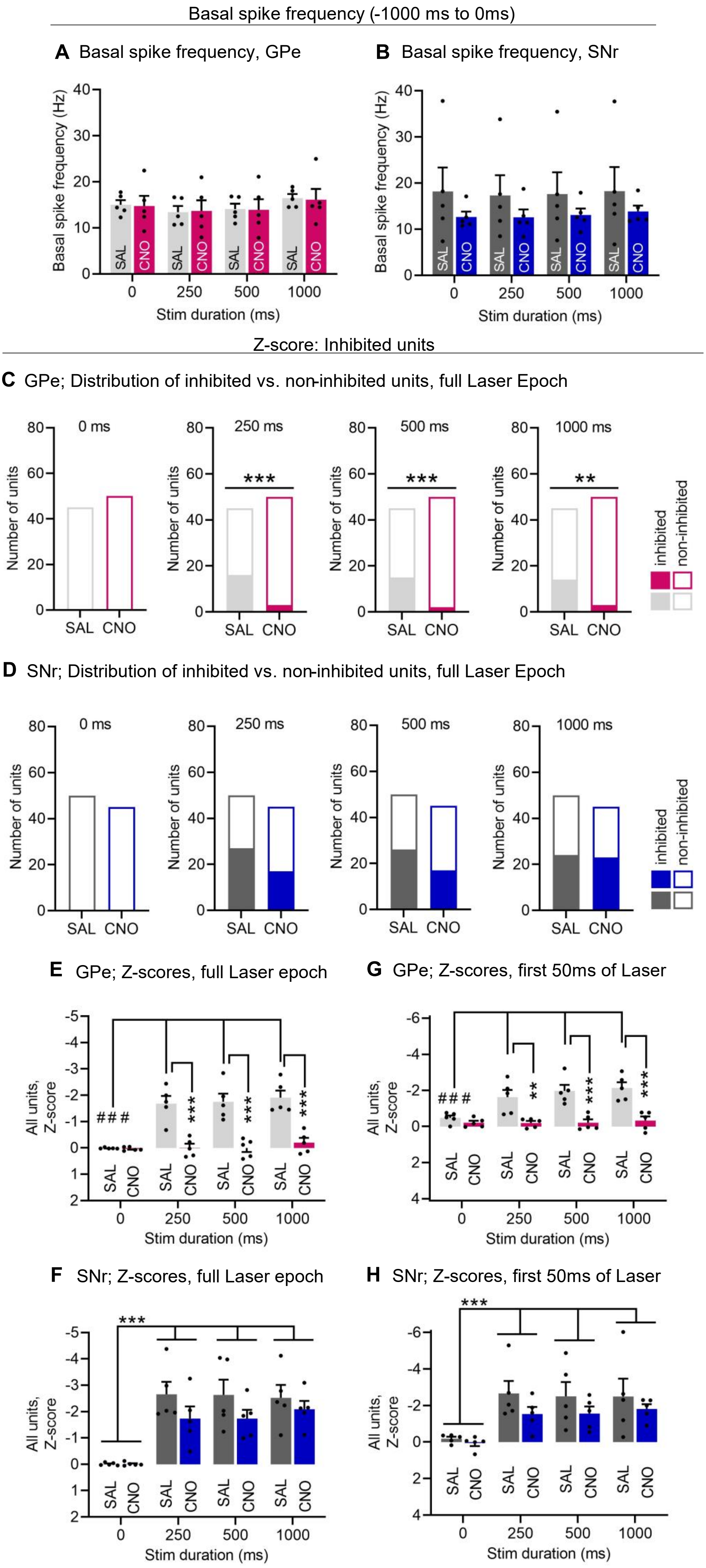
Basal spike frequency (-1000 ms to 0ms)
A Basal spike frequency, GPe
B Basal spike frequency, SNr
Basal spike frequency (Hz)
SAL
CNO
0
250
500
1000
Stim duration (ms)
Z-score: Inhibited units
C GPe; Distribution of inhibited vs. non-inhibited units, full Laser Epoch
D SNr; Distribution of inhibited vs. non-inhibited units, full Laser Epoch
0 ms
250 ms
500 ms
1000 ms
Number of units
***
**
inhibited
non-inhibited
E GPe; Z-scores, full Laser epoch
G GPe; Z-scores, first 50ms of Laser
F SNr; Z-scores, full Laser epoch
H SNr; Z-scores, first 50ms of Laser
All units, Z-score
###

Supplementary Fig. S6. Confirmation that chemogenetic manipulation of dSPN GPe terminals does not affect activity in the SNr: basal spike frequency and Z-score analysis of inhibitory responses. (Related to Fig. 6). A. Basal spike frequency in the globus pallidus externus (GPe) after chemogenetic inhibition of direct pathway striatal projection neuron (dSPN) terminals in the GPe (clozapine-N-oxide, CNO) vs. control condition (saline, SAL infusion) using the inhibitory designer receptor hM4D, calculated by averaging the spike frequency in the -1000ms to 0ms before laser optogenetic stimulation (using the activating opsin ChR2) in the various recording conditions (0, 250, 500, 1000 ms stimulation (stim) duration) (shown in Fig. 6C-F; note that in Fig.6C-F barplots have a 1ms resolution, thus the error bars (n= 5 mice) mask the individual spikes and the basal spike frequency that is now shown here in **Supplementary Fig S6A-B**) (ANOVA: stim duration x drug p=0.92, drug p=0.96, stim duration p<0.001, post-hocs SAL vs. CNO all p >0.99). **B.** Same as A. for the substantia nigra reticulata (SNr) (ANOVA: stim duration x drug p=0.60, drug p=0.37, stim duration p = 0.12). **C.** Proportion of GPe units for which the Z-score of the spike frequency is significantly decreased (inhibited units) or not (non-inhibited units) after laser stimulation at different durations calculated in the full laser epoch (Fisher's test SAL vs CNO: at 0ms p = 0.99; at 250, 500 ms: ***p<0.001; at 1000 ms: **p = 0.0024). The ratio of Inhibited units ([range] across 250,500,1000ms)/Total units recorded is: GPe-SAL: [12-17]/45; GPe-CNO: [2-3]/50. **D.** Same as **C** for the SNr (Fisher's test at all stim durations: SAL vs CNO: p = 0.99). The ratio of Inhibited units ([range] across 250,500,1000ms)/Total units recorded is: SNr-SAL: [23-28]/50; SNr-CNO: [14-23]/45. **E**. Z-score of the spike frequency in the GPe calculated in the full laser epoch for all units (ANOVA: stim duration x drug p<0.001; Sidak post-hocs SAL vs. CNO at 250, 500, 1000ms: all ***p<0.001; Sidak post-hocs 0 ms vs. other stim durations: all SAL: #p<0.001, all CNO: p=0.8-1.0). **F**. Same as **E** calculated for the SNr (ANOVA: stim duration x drug p=0.26; drug p= 0.25, main effect of stim duration: ***p<0.001; Sidak post-hoc all mice pooled: 0 ms vs. other stim durations: all ***p<0.001).). **G.** Same as **E** calculated in the first 50ms of the laser epoch (ANOVA: stim duration x drug p<0.001; Sidak post-hocs SAL vs. CNO at 250 ms **p=0.0013, 500 and 1000 ms ***p<0.001; Sidak post-hocs: SAL 0 vs. all stim durations ***p<0.001, CNO: all p=0.9-1.0). **H.** Same as **G** for the SNr (ANOVA: stim duration x drug p=0.28; drug p= 0.63, main effect of stim duration: ***p<0.001; Sidak post-hoc all mice pooled: 0 ms vs. other stim durations: all ***p<0.001). N= 5 GPe;SAL, 5 GPe;CNO, 5 SNr;SAL, 5 SNr;CNO for all panels. Data is mean±SEM. Exact p-values are given in **Supplementary Dataset S2.** Source data are provided as a Source Data file.

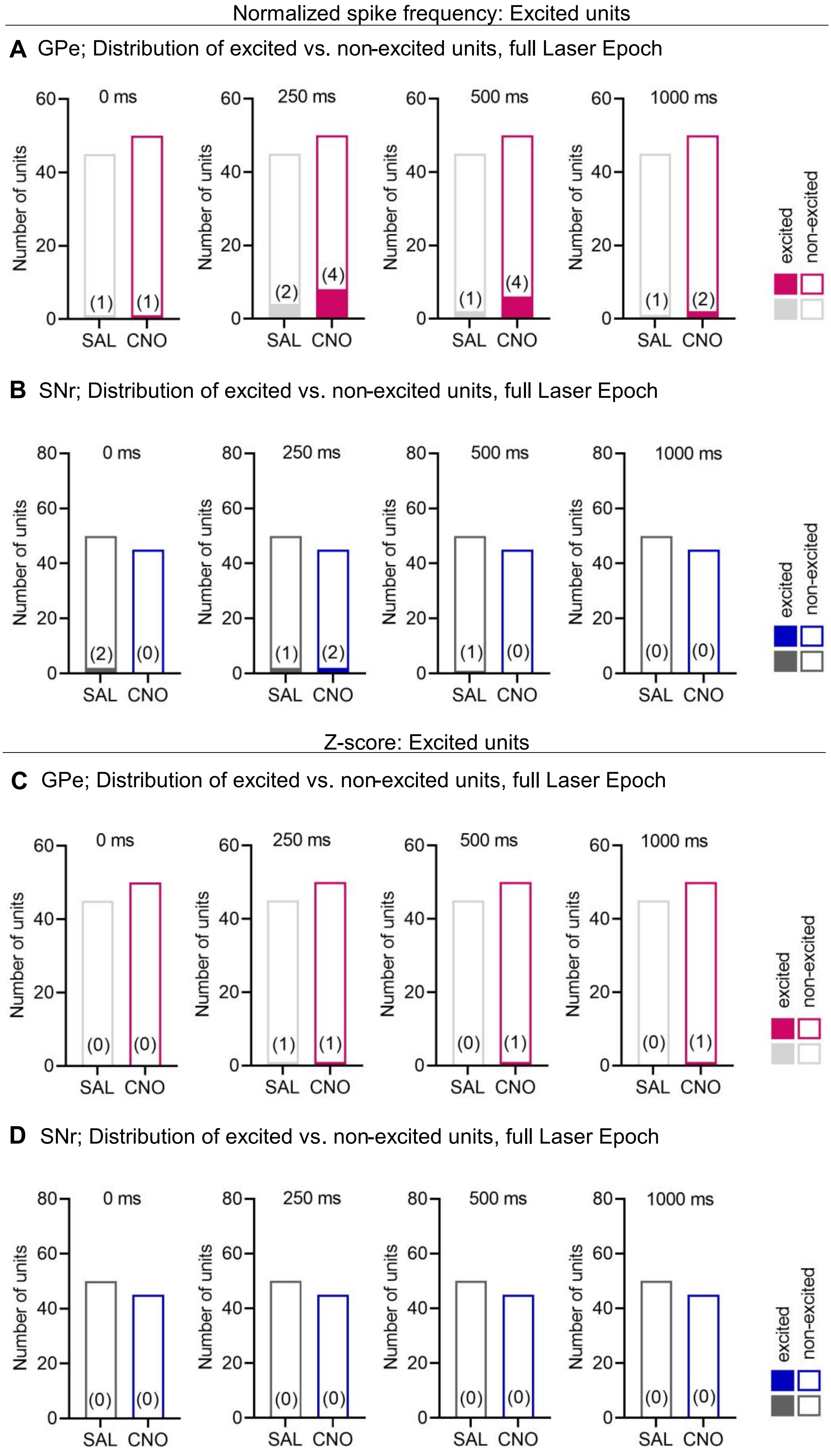

Supplementary Fig. S7. Confirmation that chemogenetic manipulation of dSPN GPe terminals does not affect activity in the SNr: Normalized spike frequency analysis and Z-score analysis of excitatory responses. (Related to Fig. 6). A. Normalized spike frequency analysis: Proportion of units in the globus pallidus externus (GPe) for which the normalized spike frequency is significantly increased (excited units) or not (non-excited units) after laser optogenetic stimulation (using the activating opsin ChR2) at different durations calculated in the full laser epoch. The ratio of Excited units ([range] across 250,500,1000ms)/Total units recorded is: control condition (saline, SAL): GPe-SAL: [1-4]/45;

experimental condition with inhibition of dSPN terminals using the inhibitory designer receptor hM4D and clozapine-N-oxide (CNO), GPe-CNO: [2-8]/50.The actual number of excited units found is labeled in the bar graphs in parenthesis. **B.** Same as **A** for the SNr. The ratio of Excited units ([range] across 250,500,1000ms)/Total units is: SNr-SAL: [0-3]/50; SNr-CNO: [0-2]/45. The actual number of excited units found is labeled in the bar graphs in parenthesis. **C.** Z-score analysis: Proportion of units in the GPe for which the Z-score of the spike frequency is significantly increased (excited units) or not (non-excited units) after laser stimulation at different durations calculated in the full laser epoch. The ratio of Excited units ([range] across 250,500,1000ms)/Total units recorded is: GPe-SAL: [0-1]/45; GPe-CNO: [0-1]/50. The actual number of excited units found is labeled in the bar graphs in parenthesis. **F.** Same as **G** for the SNr. The ratio of Excited units ([range] across 250,500,1000ms)/Total units is: SNr-SAL: [0]/50; SNr-CNO: [0]/45. The actual number of excited units found is labeled in the bar graphs in parenthesis. The number of excited units across conditions is too low for statistical analysis. Mice: N= 5 GPe;SAL, 5 GPe;CNO, 5 SNr;SAL, 5 SNr;CNO for all panels. Data is mean±SEM. Exact p-values are given in **Supplementary Dataset S2.** dSPN: direct pathway striatal projection neuron. Source data are provided as a Source Data file.

A dSPN terminal inhibition in the GPe with optogenetics: lack of effect on center vs. periphery locomotion

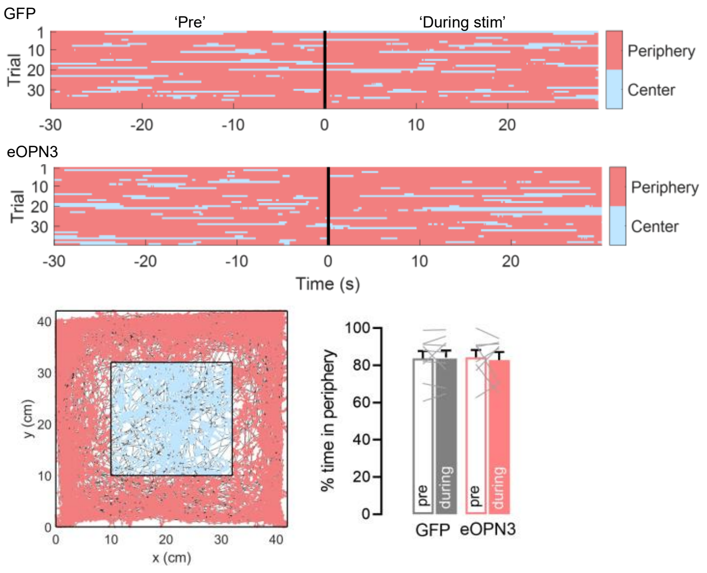

Supplementary Fig. S8. Optogenetic inhibition of dSPN terminals in the GPe does not affect anxiety (Related to Fig. 7) A. Top: Heatmaps showing behavioral classification of individual videoframes into center or periphery zones of the open field, before (pre) and during laser stimulation (stim) across trials (all animals), i.e. during inhibition of direct pathway striatal projection neuron (dSPN) terminals in the globus pallidus externus (GPe) using the inhibitory moskito rhodopsin eOPN3. Bottom, left: Representative trajectory of 1 animal's body center in the open field, separated by center (blue) and periphery (pink) zones. Bottom, right: Quantification: optogenetic inhibition of dSPN terminals in the GPe does not affect percent time spent in the periphery vs. center (ANOVA: virus x epoch p=0.79), suggesting absence of anxiety effects. N= 8 eOPN3, 9 GFP. Data is mean±SEM. Exact p-values are given in **Supplementary Dataset S2.** Source data are provided as a Source Data file.

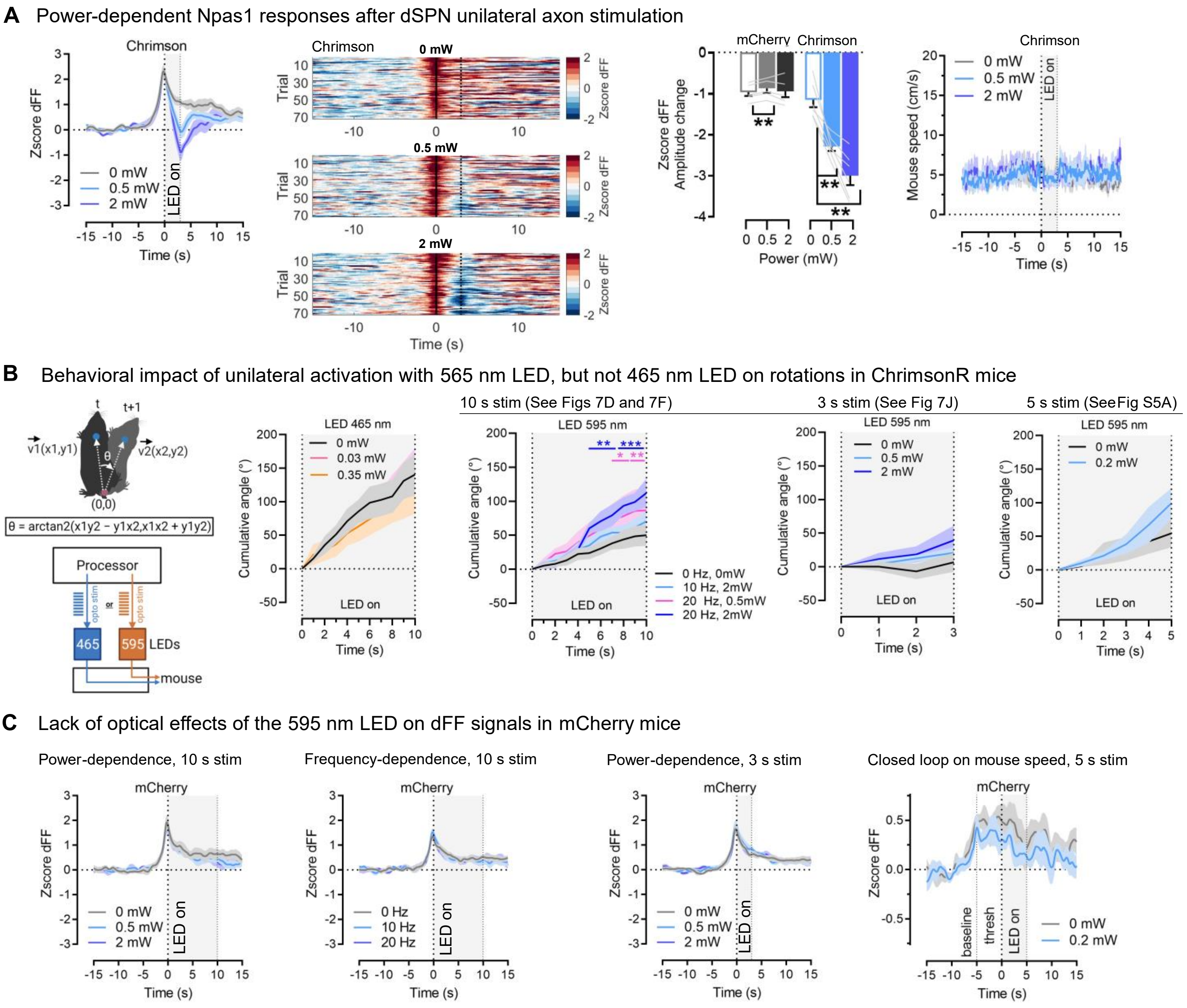

Supplementary Fig. S9. Additional data and controls for the all-optical setup for optogenetic stimulation of dSPN axons and Npas1 calcium imaging (GCaMP6s) in the GPe (Related to Fig. 8). A. 20 Hz optogenetic stimulation of direct pathway striatal projection neuron (dSPN) axons in the globus pallidus externus (GPe) using the activating opsin ChrimsonR for 3-sec (0, 0.5 or 2 mW) leads to a power-dependent reduction in Npas1 calcium activity (measured with the calcium indicator GCaMP6s) in ChrimsonR mice without affecting mouse speed. Left: Average traces, Middle: Heatmaps of all trials, Right: Amplitude change in Npas1 normalized fluorescence i.e. deltaF/F (dFF) in the optogenetic window (ANOVA: virus x power p<0.001; post-hocs **p<0.01). Far right: Mouse speed over time (ANOVA: power p=0.59) **B.** Left, top: Formula for the calculation of the angle (degree) during mouse rotation, using positions at two consecutive timepoints (t, t+1). The position vectors v1 and v2 are calculated using x1,y1 and x2,y2 positions of the head, normalized to the position of the lower body (0,0). Left, bottom: optogenetic stimulation (stim) at 465 nm (= LED for photometry imaging) or 595 nm (=spectrum for ChrimsonR activation). Right: Unilateral light stimulation in Drd1-cre mice with 595 nm LED (see parameters below), but not 465 nm LED (20Hz, 10s, 0.03 or 0.35 mW; ANOVA: power p=0.85, power x timepoints p=0.98) leads to rotational behavior. The 465 nm LED was tested at 0.03 mW (=photometry recording power) and at its maximal output (0.35 mW, >10x higher than photometry power). This indicates that the 465 nm does not interfere with ChrimsonR-dependent effects on behavior, i.e. it does not reliably activate ChrimsonR molecules. 595 nm-induced rotations are detected with strong 10 s stimulation protocols (10 s/20Hz/0.5mW and 10 s/20Hz/2mW) but not milder ones (10 s/10Hz/2mW) (ANOVA: stim parameters p=0.41, stim parameters x timepoints p=0.04; Sidak post-hocs tests are comparisons to the control trials 0Hz/0mW at specific timepoints *p<0.05, **p<0.01 and ***p<0.001). No significant rotations were detected with 3s stimulation protocols (3

s/20Hz at 0.5 or 2mW) (ANOVA: power p=0.32, power x timepoints p=0.28) or with 5s/ultra-low power (5 s/20Hz at 0.2 mW) (ANOVA: power p=0.58, power x timepoints p=0.42). This suggests that the effects of dSPN stimulation on Npas1 ativity could not be solely explained by changes in mouse behavior. **C.** Light stimulation with 595 nm LED in Drd1-cre mice expressing mCherry does not affect Npas1 dFF as shown on average dFF traces, i.e. it does not reliably excite GCaMP molecules This indicates that the 595 nm does not interfere with Npas1 photometry recording in the absence of ChrimsonR expression. Heatmaps, straight line = LED onset, dashed line = LED off. N= 5 mCherry, N=6 ChrimsonR throughout. Data is mean±SEM. Exact p-values are given in **Supplementary Dataset S2.** Source data are provided as a Source Data file.

A Neuroanatomical map of implant locations (1/2)

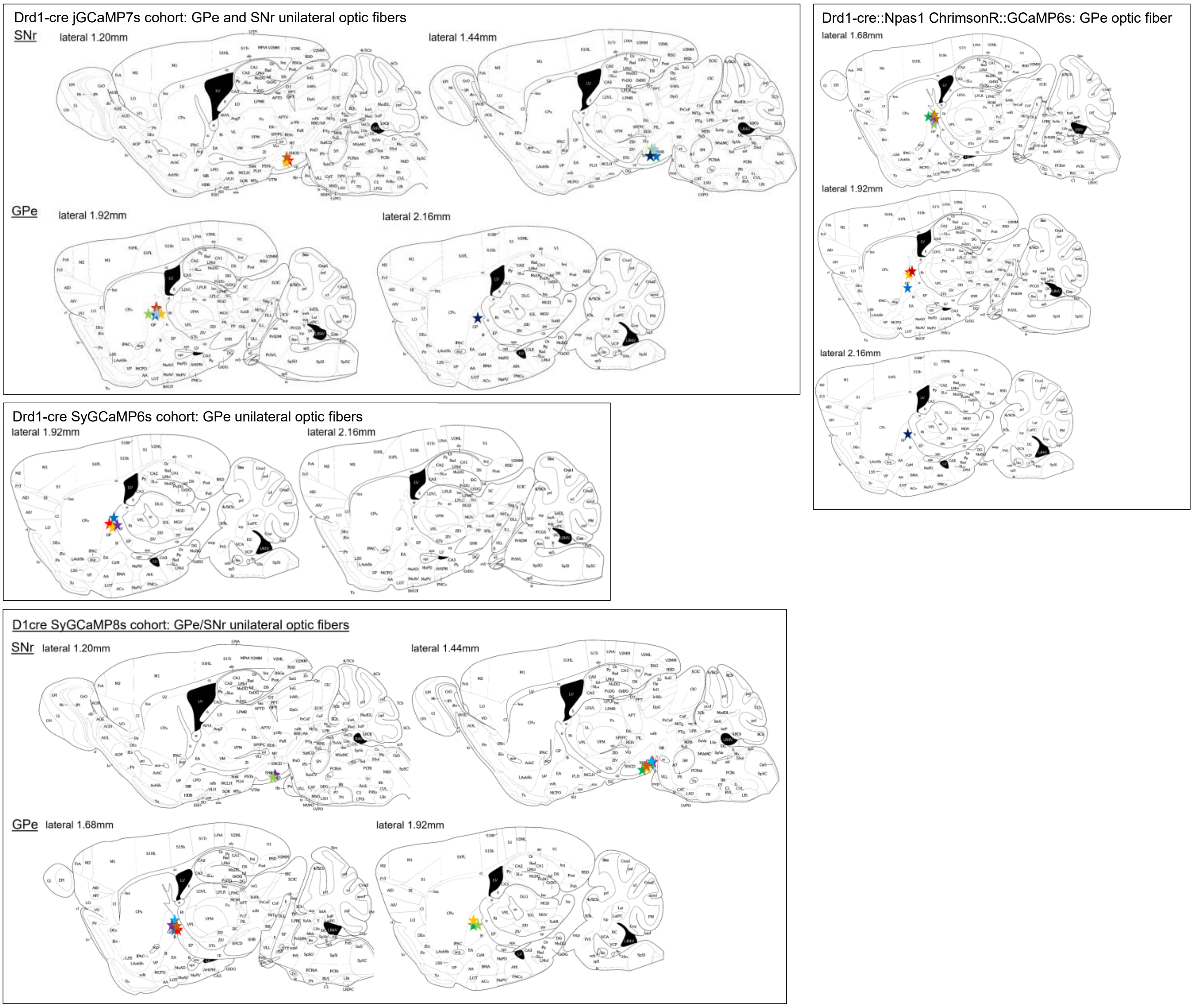

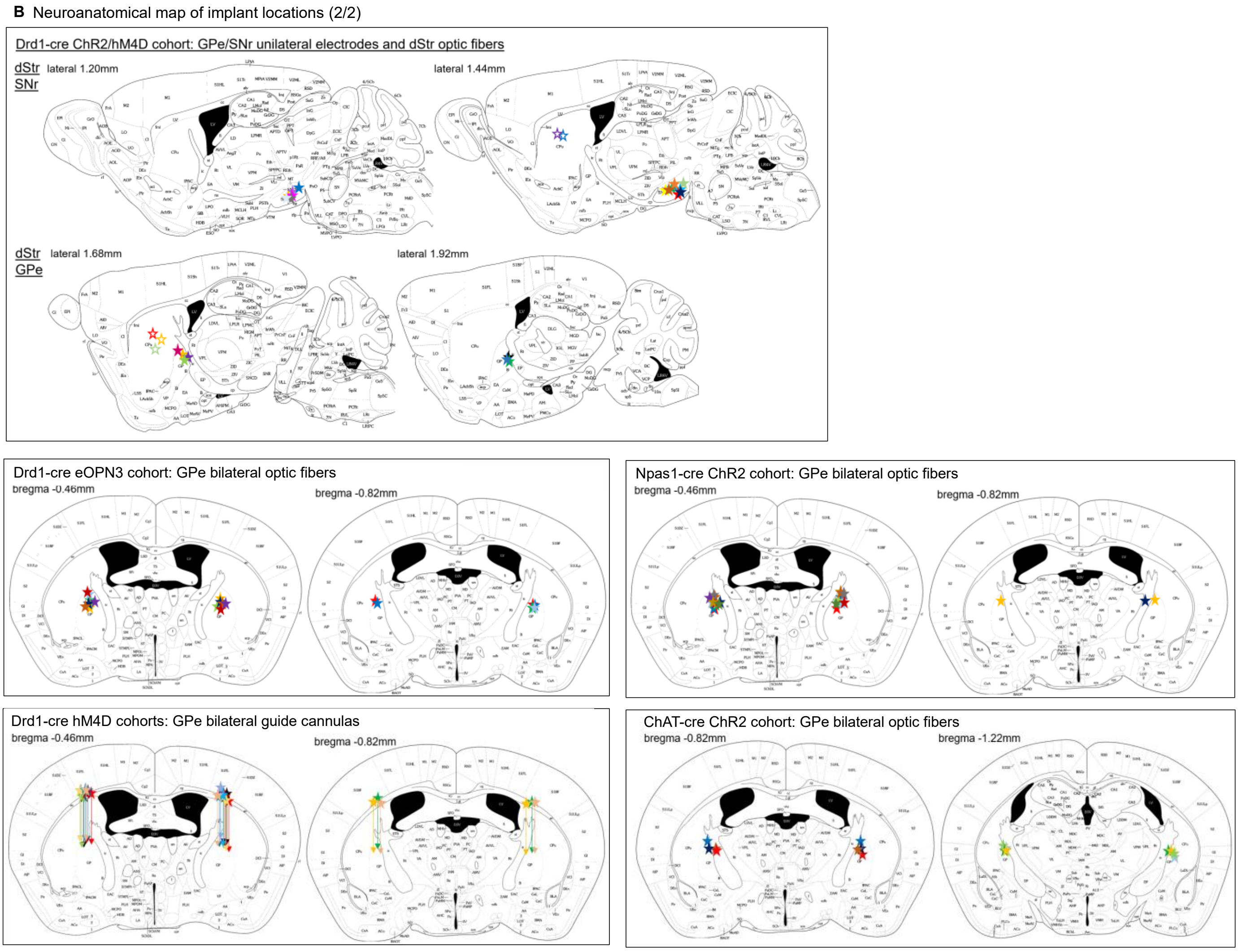

Supplementary Fig. S10. Summary of implant locations (optic fibers or cannulas) into the dStr, GPe or SNr (Related to Fig. 2-9 and Supplementary Fig S4-S9). Implant location for each animal is represented with a star of a different color. Individual cohorts are shown in separate rectangles. For Drd1-cre ChR2/hM4D animals receiving acute optic fibers for in vivo physiology recordings, optic fiber tracts were not always clearly distinguishable as they were only acutely inserted; only brains with clear paths have the dStr optic fiber location depicted here. Since the globus pallidus externus (GPe) and substantia nigra reticulata (SNr) were scanned at different locations for physiology, glass electrodes paths could be observed at different anterior-posterio (AP) levels in some brains; for clarity, the most clear electrode location is shown here. ChR2: channel-rhodopsin 2 (optogenetic activating ion channel), ChrimsonR: red-shifted optogenetic activating ion channel, dStr: dorsal striatum, eOPN3: enhanced mosquito homolog of the vertebrate encephalopsin (optogenetic silencing rhodopsin), GCaMP7s/GCaMP6s: calcium sensors, hM4D: inhibitory muscarinic M4 receptor–based Gi-coupled DREADD (designer receptor exclusively activated by designer drug), SyGCaMP6s: Synaptophysin-GCaMP6s.

A. Auto-correlation of raw data: GCaMP7s vs. SyGCaMP8s

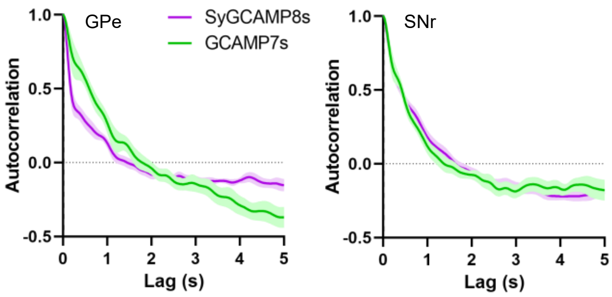

B. GCaMP7s: Comparison raw data vs. data filtered with a 0.5s moving average smoothing

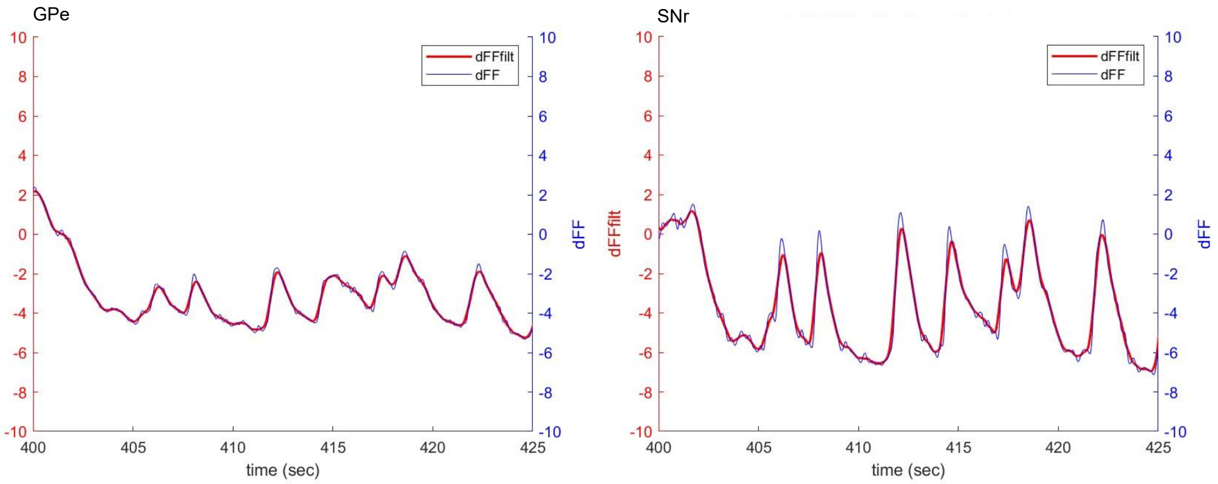

C. GCaMP7s: Peak detection with raw data vs. with a 0.5s moving average smoothing

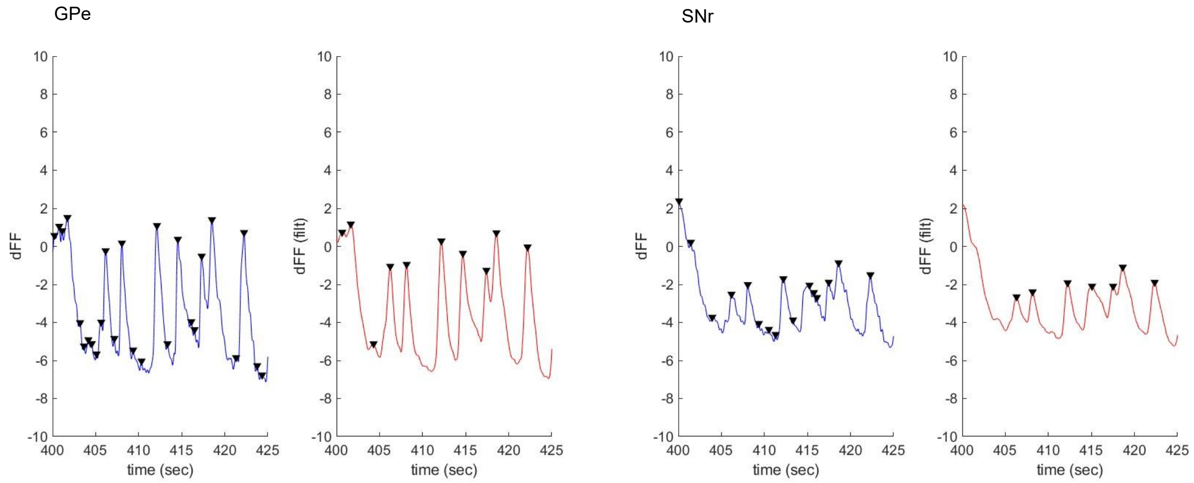

Supplementary Fig. S11. Verifications for data analysis of in vivo calcium recordings (fiber photometry) of direct pathway striatal projection neuron (dSPN) terminals in the globus pallidus externus (GPe) and substantia nigra (SNr). A. Autocorrelation analysis of GCaMP7s and Synaptophysin-GCaMP8s (SyGCaMP8s) done on data acquired with the photometry processor (3Hz low-pass online; no offline low-pass filter). This was done to address the possibility that SyGCaMP8s may be under-reporting the difference between GPe and SNr terminal photometry signals shown in **Fig. 4G** as compared to data shown with jGCaMP7s (**Fig. 3I**). This is unlikely since jGCaMP8s has substantially faster on and off kinetics (half rise-time: 21 ms; half-decay time: 52 ms) than jGCaMP7s (half rise-time: 67 ms; half-decay time: 81 ms) (PMID 36922596). Since we added a targeting motif (synaptophysin) to the GCaMP8s, the kinetics may be slightly different. To address this, we analyzed the autocorrelation of the in vivo calcium signal for SyGCaMP8s and GCaMP7s as a proxy for kinetics. An autocorrelation that quickly decreases when increasing the lag (seconds) potentially reflects a signal with more fast fluctuations (potentially meaningful, potentially noise), and in principle, faster kinetics. This analysis was done at baseline (selected as epochs when animal speed in an open field was <3cm/s). We find that the autocorrelation for SyGCaMP8s decreased faster at increasing lags compared to GCaMP7s; this was evident in the GPe (not SNr) likely due to the fact that there are less synapses and therefore lower signal to noise ratio.

This is consistent with the faster kinetics of jGCaMP8s. Therefore, it is unlikely that SyGCAMP8s is undereporting the difference between GPe and SNr terminal photometry signals. **B-C.** Comparison between raw and 0.5s smoothed data (in a moving window) of normalized fluorescence, i.e. deltaF/F (dFF or ΔF/F) data obtained with GCaMP7s. B: Raw (blue) and filtered (red) data overlay and C: Raw (blue) and filtered (red) data peak detection. This is the only dSPN GCAMP data where we kept an offline filter (smoothing in 0.5 s moving average window) as shown in **Supplementary Fig. S4D-E** where we quantify peak frequency, amplitude or interpeak interval. Here it was important to apply a filter to avoid overcounting high-frequency peaks that poorly track the behavior of interest (jumps). For instance, one can see in **Fig. 3F** that relevant behavioral transients (jumps) are 0.5 to 1.5 sec long. This is consistent with data computed in **Supplementary Fig.S4D-E** where for example we count interpeak intervals of 0.5 to 4 sec. This smoothing does not alter the data, as shown here.

Supplementary Note 1. Sequence for AAV9-CAG-DIO-Synaptophysin-jGCaMP8s. Synaptophysin was cloned into a vector expressing the calcium indicator jGCaMP8s in a cre-dependent manner (lox P sites) under the CAG promoter. Map and full sequence are provided below.

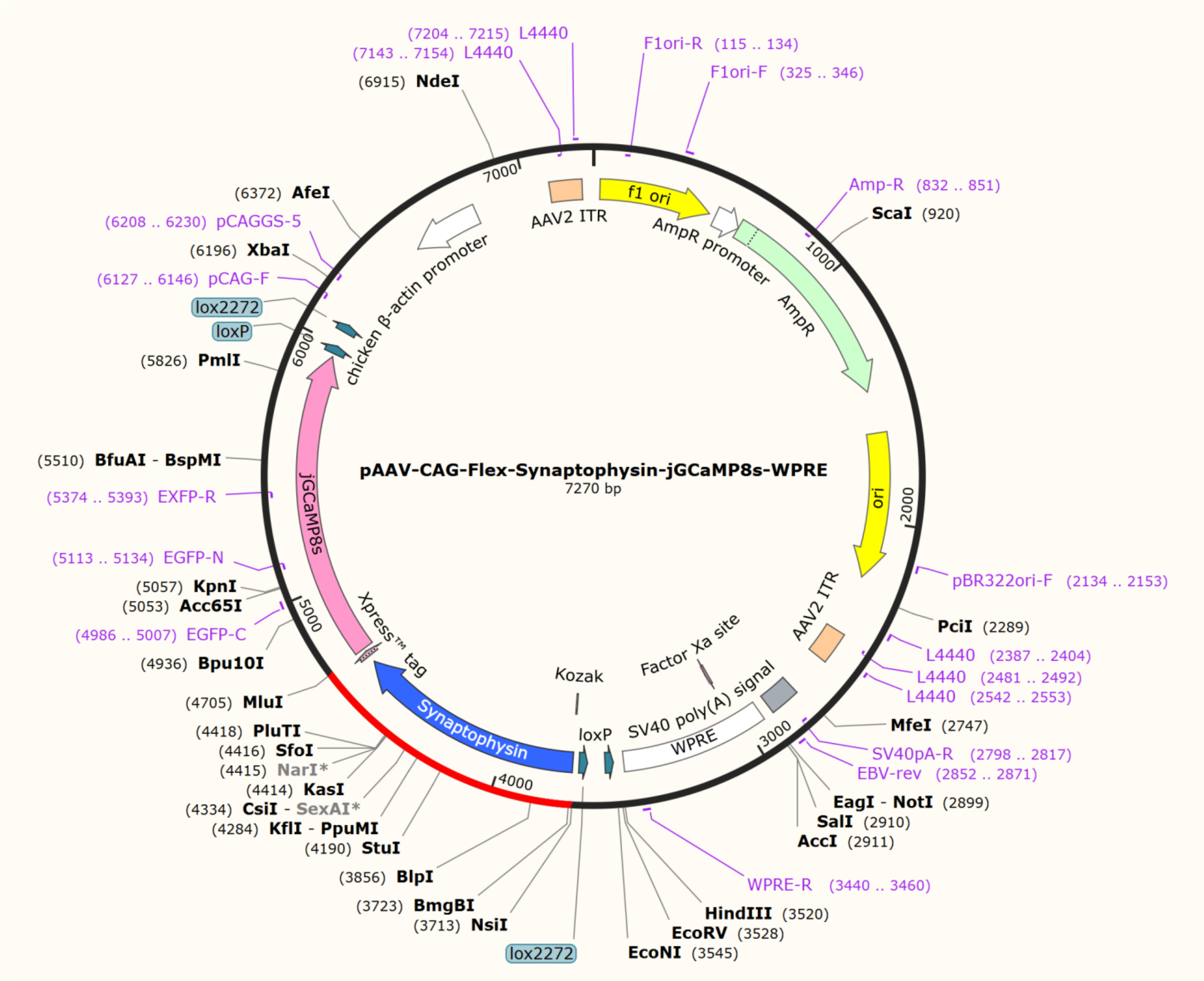

agttgcgcagcctgaatggcgaatgggacgcgccctgtagcggcgcattaagcgcggcgggtgtggtggttacgcgcagcgtgaccgctacacttgccagcgccct
agcgcccgctcctttcgctttcttcccttcctttctcgccacgttcgccggctttccccgtcaagctctaaatcgggggctccctttagggttccgatttagtgctttacggcacct
cgaccccaaaaaacttgattagggtgatggttcacgtagtgggccatcgccctgatagacggtttttcgccctttgacgttggagtccacgttctttaatagtggactcttgtt
ccaaactggaacaacactcaaccctatctcggtctattcttttgatttataagggattttgccgatttcggcctattggttaaaaaatgagctgatttaacaaaaatttaacgc
gaattttaacaaaatattaacgcttacaatttaggtggcacttttcggggaaatgtgcgcggaacccctatttgtttatttttctaaatacattcaaatatgtatccgctcatgag
acaataaccctgataaatgcttcaataatattgaaaaaggaagagtatgagtattcaacatttccgtgtcgcccttattcccttttttgcggcattttgccttcctgtttttgctcac
ccagaaacgctggtgaaagtaaaagatgctgaagatcagttgggtgcacgagtgggttacatcgaactggatctcaacagcggtaagatccttgagagttttcgcccc
gaagaacgttttccaatgatgagcacttttaaagttctgctatgtggcgcggtattatcccgtattgacgccgggcaagagcaactcggtcgccgcatacactattctcag
aatgacttggttgagtactcaccagtcacagaaaagcatcttacggatggcatgacagtaagagaattatgcagtgctgccataaccatgagtgataacactgcggcc
aacttacttctgacaacgatcggaggaccgaaggagctaaccgcttttttgcacaacatgggggatcatgtaactcgccttgatcgttgggaaccggagctgaatgaag
ccataccaaacgacgagcgtgacaccacgatgcctgtagcaatggcaacaacgttgcgcaaactattaactggcgaactacttactctagcttcccggcaacaatta
atagactggatggaggcggataaagttgcaggaccacttctgcgctcggcccttccggctggctggtttattgctgataaatctggagccggtgagcgtgggtctcgcgg
tatcattgcagcactggggccagatggtaagccctcccgtatcgtagttatctacacgacggggagtcaggcaactatggatgaacgaaatagacagatcgctgagat
aggtgcctcactgattaagcattggtaactgtcagaccaagtttactcatatatactttagattgatttaaaacttcatttttaatttaaaaggatctaggtgaagatcctttttgat
aatctcatgaccaaaatcccttaacgtgagttttcgttccactgagcgtcagaccccgtagaaaagatcaaaggatcttcttgagatcctttttttctgcgcgtaatctgctgct

tgcaaacaaaaaaaccaccgctaccagcggtggtttgtttgccggatcaagagctaccaactctttttccgaaggtaactggcttcagcagagcgcagataccaaata
ctgttcttctagtgtagccgtagttaggccaccacttcaagaactctgtagcaccgcctacatacctcgctctgctaatcctgttaccagtggctgctgccagtggcgataag
tcgtgtcttaccgggttggactcaagacgatagttaccggataaggcgcagcggtcgggctgaacggggggttcgtgcacacagcccagcttggagcgaacgacct
acaccgaactgagatacctacagcgtgagctatgagaaagcgccacgcttcccgaagggagaaaggcggacaggtatccggtaagcggcagggtcggaacag
gagagcgcacgagggagcttccagggggaaacgcctggtatctttatagtcctgtcgggtttcgccacctctgacttgagcgtcgatttttgtgatgctcgtcaggggggc
ggagcctatggaaaaacgccagcaacgcggcctttttacggttcctggccttttgctggccttttgctcacatgttctttcctgcgttatcccctgattctgtggataaccgtatt
accgcctttgagtgagctgataccgctcgccgcagccgaacgaccgagcgcagcgagtcagtgagcgaggaagcggaagagcgcccaatacgcaaaccgcctc
tccccgcgcgttggccgattcattaatgcagctgcgcgctcgctcgctcactgaggccgcccgggcaaagcccgggcgtcgggcgacctttggtcgcccggcctcagt
gagcgagcgagcgcgcagagagggagtggccaactccatcactaggggttccttgtagttaatgattaacccgccatgctacttatctacgtagccatgctctaggaa
gatccttatcgattttaccacatttgtagaggttttacttgctttaaaaaacctcccacatctccccctgaacctgaaacataaaatgaatgcaattgttgttgttaacttgtttatt
gcagcttataatggttacaaataaagcaatagcatcacaaatttcacaaataaagcatttttttcactgcattctagttgtggtttgtccaaactcatcaatgtatcttatcatgt
ctgctcgaagcggccgcccgggtcgacggtatcgatgcggggaggcggcccaaagggagatccgactcgtctgagggcgaaggcgaagacgcggaagaggc
cgcagagccggcagcaggccgcgggaaggaaggtccgctggattgagggccgaagggacgtagcagaaggacgtcccgcgcagaatccaggtggcaacac
aggcgagcagccaaggaaaggacgatgatttccccgacaacaccacggaattgtcagtgcccaacagccgagcccctgtccagcagcgggcaaggcaggcgg
cgatgagttccgccgtggcaatagggagggggaaagcgaaagtcccggaaaggagctgacaggtggtggcaatgccccaaccagtgggggttgcgtcagcaaa
cacagtgcacaccacgccacgttgcctgacaacgggccacaactcctcataaagagacagcaaccaggatttatacaaggaggagaaaatgaaagccatacgg
gaagcaatagcatgatacaaaggcattaaagcagcgtatccacatagcgtaaaaggagcaacatagttaagaataccagtcaatctttcacaaattttgtaatccaga
ggttgattatcgataagcttgatatcgaattcgaacctcttcgagggacataacttcgtataatgtatgctatacgaagttatgggtcgatggtgagatctggactagagggt
cgatggtgatgcttggatcaaaataggaagaatcccaatgataacttcgtataaagtatcctatacgaagttatactaggatccgccaccatgcaTACCatggacgtg
gtgaatcagctggtggctgggggtcagttccgggtggtcaaggagccccttggcttcgtgaaggtgctgcagtgggtctttgccatcttcgcctttgctacgtgtggcagct
acaccggggagcttcggctgagcgtggagtgtgccaacaagacggagagtgccctcaacatcgaagttgaattcgagtaccccttcaggctgcaccaagtgtacttt
gatgcaccctcctgcgtcaaaggggggcactaccaagatcttcctggttggggactactcctcgtcggctgaattctttgtcaccgtggctgtgtttgccttcctctactccatg
gggggccctggccacctacatcttcctgcagaacaagtaccgagagaacaacaaagggcctatgatggactttctggctacagccgtgttcgctttcatgtggctagttag
ttcatcagcctgggccaaaggcctgtccgatgtgaagatggccacggacccagagaacattatcaaggagatgcccatgtgccgccagacagggaacacatgcaa
ggaactgagggaccctgtgacttcaggactcaacaccacagtggtgtttggcttcctgaacctggtgctctgggttggcaacttatggttcgtgttcaaggagacaggctg
ggcagccccattcatgcgcgcacctccaggcgccccggaaaagcaaccagcacctggcgatgcctacggcgatgcgggctacgggcagggccccggaggctat
gggccccaggactcctacgggcctcagggtggttatcaacccgattacgggcagccagccagcggtggcggtggctacgggcctcagggcgactatgggcagca
aggctatggccaacagggtgcgcccacctccttctccaatcaggatccaccgggactggtggacagcaaatgggtcgggatctgtacgacgatgacgataaggatct
cgccaAcgcgtcgcaagaagaccttcaaggaggtggccaccgccgtgaagatcatcgccatgctgatgggcctgaagatcaacgtctatatcaaggccgacaagc
agaagaacggcatcaaggcgaacttccacatccgccacaacatcgaggacggcggcgtgcagctcgcctaccactaccagcagaacacccccatcggcgacg
gccccgtgctgctgcccgacaaccactacctgagcgtggagtccaaactttcgaaagaccccaacgagaagcgcgatcacatggtcctgctggagttcgtgaccgc
cgccgggatcactctcggcatggacgagctgtacaagggcggtaccggagggagcatggtgagcaagggcgaggagctgttcaccggggtggtgcccatcctggt
cgagctggacggcgacgtaaacggccacaagttcagcgtgtccggcgagggtgagggcgatgccacctacggcaagctgaccctgaagttcatctgcaccaccg
gcaagctgcccgtgccctggcccaccctcgtgaccaccctgacctacggcgtgcagtgcttcagccgctaccccgaccacatgaagcagcacgacttcttcaagtcc
gccatgcccgaaggctacatccaggagcgcaccatcttcttcaaggacgacggcaactacaagacccgcgccgaggtgaagttcgagggcgacaccctggtgaa
ccgcatcgagctgaagggcatcgacttcaaggaggacggcaacatcctggggcacaagctggagtacaacctgccggaccaactgactgaagagcagatcgca
gaatacaaagaggctttctccctatttgacaaggacggggatgggacaataacaaccaaggagctggggacggtgatgcggtctctcggacacaaccccacagaa
gcagagctgcaggacatgatcaatgaagtagatgccgacggtgacggcacaatcgacttccctgagttcctgacaatgatggcaagaaaaatgaaatacagggac
acggaagaagaaattagagaagcgttcggtgtgtttgataaggatggcaatggctacatcagtgcagcagagcttcgccacgtgatgacaaaccttggagagaagtt
aacagatgaagaggttgatgaaatgatcagggaagcagacatcgatggggatggtcaggtaaactacgaagagtttgtacaaatgatgacagcgaagtgaaagct
ctagtataacttcgtatagcatacattatacgaagttatgggtcgatggtgatgcttggcaattcgggtcgatggtgaagcattggtcttcctattttgatataacttcgtatagg
atactttatacgaagttatggggatccaattctttgccaaaatgatgagacagcacaataaccagcacgttgcccaggagctgtaggaaaaagaagaaggcatgaac
atggttagcagaggctctagagccgccggtcacacgccagaagccgaaccccgccctgccccgtcccccccgaaggcagccgtcccccgcggacagccccga
gccgctcccccgcacaaagggccctcccggagcccctcaaggctttcacgcagccacagaaaagaaacaagccgtcattaaaccaagcgctaattacagcccg
gaggagaagggccgtcccgcccgctcacctgtgggagtaacgcggtcagtcagagccggggcgggcggcgcgaggcggcggcggagcggggcacggggcg
aaggcagcgcgcagcgactcccgcccgccgcgcgcttcgctttttatagggccgccgccgccgccgcctcgccataaaaggaaactttcggagcgcgccgctctga

ttggctgccgccgcacctctccgcctcgccccgccccgcccctcgccccgccccgccccsccyggcgcgcgccccccccccccccccgcccccatcgctgcacaa
aataattaaaaaataaataaatacaaaattgggggtggggagggggggggagatggggagagtgaagcagaacgtggggctcacctcgaccatggtaatagcgat
gactaatacgtagatgtactgccaagtaggaaagtcccataaggtcatgtactgggcataatgccaggcgggccatttaccgtcattgacgtcaatagggggcgtactt
ggcatatgatacacttgatgtactgccaagtgggcagtttaccgtaaatactccacccattgacgtcaatggaaagtccctattggcgttactatgggaacatacgtcatta
ttgacgtcaatggtactcttcctagagcatggctacgtagataagtagcatggcgggttaatcattaactacaaggaacccctagtgatggagttggccactccctctctg
cgcgctcgctcgctcactgaggccgggcgaccaaaggtcgcccgacgcccgggctttgcccgggcggcctcagtgagcgagcgagcgcgcagctggcgtaatag
cgaagaggcccgcaccgatcgcccttcccaac
