# Supplementary material for: A non-canonical striatopallidal Go pathway that supports motor control
Source: Nat Commun. 2023 Oct 23;14:6712. doi: 10.1038/s41467-023-42288-1 (PMC10593790; doi:10.1038/s41467-023-42288-1)
Supplement: Supplementary file 3 — Description of Additional Supplementary Files [file 41467_2023_42288_MOESM3_ESM.pdf]

Description of Additional Supplementary Files

Supplementary Dataset 1. Summary table giving list and reference for all reagents and equip ment used

Supplementary Dataset 2. Summary table giving statistical analyses and results for each Figure

Supplementary Video 1. dSPN terminal calcium dynamics in the GPe and SNr and mouse speed during open field locomotion. A cre-dependent jGCaMP7s calcium indicator was used in Drd1-cre mice. Mouse speed is smoothed in 2-sec bins.

Supplementary Video 2. dSPN terminal calcium dynamics in the GPe and SNr and mouse behavior during a rotarod task. A cre-dependent jGCaMP7s calcium indicator was used in Drd1-cre mice. Position of the mouse's lower body in the Y axis allows to track jumps during running.

Supplementary Video 3. Representative video showing mouse speed before and after optogenetic inhibition of dSPN terminals in the GPe in an open field. The cre-dependent eOPN3 inhibitory opsin was used in Drd1-cre mice.

Supplementary Video 4. Representative video showing GPe Npas1 calcium dynamics and mouse speed before and after optogenetic stimulation of dSPN GPe axons in an open field. Cre-dependent GCaMP6s calcium indicator and ChrimsonR activating opsin were used in Drd1-cre;Npas1-cre mice. Mouse speed is smoothed in 2-sec bins.
