# Supplementary material for: A non-canonical striatopallidal Go pathway that supports motor control
Source: Nat Commun. 2023 Oct 23;14:6712. doi: 10.1038/s41467-023-42288-1 (PMC10593790; doi:10.1038/s41467-023-42288-1)
Supplement: Supplementary file 10 — Reporting Summary [file 41467_2023_42288_MOESM10_ESM.pdf]

nature portfolio

Corresponding author(s): Christoph Kellendonk, Marie A. Labouesse

Last updated by author(s): Sep 15, 2023

# Reporting Summary

Nature Portfolio wishes to improve the reproducibility of the work that we publish. This form provides structure for consistency and transparency in reporting. For further information on Nature Portfolio policies, see our Editorial Policies and the Editorial Policy Checklist.

## Statistics

For all statistical analyses, confirm that the following items are present in the figure legend, table legend, main text, or Methods section.

| n/a | Confirmed | |
|---|---|---|
| ☐ | ☒ | The exact sample size (*n*) for each experimental group/condition, given as a discrete number and unit of measurement |
| ☐ | ☒ | A statement on whether measurements were taken from distinct samples or whether the same sample was measured repeatedly |
| ☐ | ☒ | The statistical test(s) used AND whether they are one- or two-sided<br>*Only common tests should be described solely by name; describe more complex techniques in the Methods section.* |
| ☐ | ☒ | A description of all covariates tested |
| ☐ | ☒ | A description of any assumptions or corrections, such as tests of normality and adjustment for multiple comparisons |
| ☐ | ☒ | A full description of the statistical parameters including central tendency (e.g. means) or other basic estimates (e.g. regression coefficient) AND variation (e.g. standard deviation) or associated estimates of uncertainty (e.g. confidence intervals) |
| ☐ | ☒ | For null hypothesis testing, the test statistic (e.g. *F*, *t*, *r*) with confidence intervals, effect sizes, degrees of freedom and *P* value noted<br>*Give P values as exact values whenever suitable.* |
| ☒ | ☐ | For Bayesian analysis, information on the choice of priors and Markov chain Monte Carlo settings |
| ☒ | ☐ | For hierarchical and complex designs, identification of the appropriate level for tests and full reporting of outcomes |
| ☐ | ☒ | Estimates of effect sizes (e.g. Cohen's *d*, Pearson's *r*), indicating how they were calculated |

*Our web collection on statistics for biologists contains articles on many of the points above.*

## Software and code

Policy information about availability of computer code

| | |
|---|---|
| Data collection | Anymaze (Stoelting), v6.34; Synapse (TDT), v95; Neuroscope, v2. |
| Data analysis | DeepLabCut, v2.1.8.2; Fiji (ImageJ), v1.53, GraphPad Prism, v8.2.1; Matlab (Mathworks), vR2019a. Custom-written code are deposited at Figshare https://doi.org/10.6084/m9.figshare.23609595.v2 |

For manuscripts utilizing custom algorithms or software that are central to the research but not yet described in published literature, software must be made available to editors and reviewers. We strongly encourage code deposition in a community repository (e.g. GitHub). See the Nature Portfolio guidelines for submitting code & software for further information.

## Data

Policy information about availability of data

All manuscripts must include a data availability statement. This statement should provide the following information, where applicable:

- Accession codes, unique identifiers, or web links for publicly available datasets
- A description of any restrictions on data availability
- For clinical datasets or third party data, please ensure that the statement adheres to our policy

Source data are provided with this paper. DeepLabCut datasets are deposited at Zenodo (https://doi.org/10.5281/zenodo.6448813 and https://doi.org/10.5281/zenodo.6448595). Original codes are deposited at Figshare (https://doi.org/10.6084/m9.figshare.23609595.v3). Any additional information or raw datasets required to reanalyze the data reported in this paper is available from the lead contact upon request.

## Human research participants

Policy information about studies involving human research participants and Sex and Gender in Research.

| | |
|---|---|
| Reporting on sex and gender | N/A |
| Population characteristics | N/A |
| Recruitment | N/A |
| Ethics oversight | N/A |

Note that full information on the approval of the study protocol must also be provided in the manuscript.

# Field-specific reporting

Please select the one below that is the best fit for your research. If you are not sure, read the appropriate sections before making your selection.

[x] Life sciences    [ ] Behavioural & social sciences    [ ] Ecological, evolutionary & environmental sciences

For a reference copy of the document with all sections, see nature.com/documents/nr-reporting-summary-flat.pdf

# Life sciences study design

All studies must disclose on these points even when the disclosure is negative.

| | |
|---|---|
| Sample size | No sample-size calculation was performed. Sample size was determined based on our previous experiments done using the same techniques where power was sufficient (see in particular Gallo et al., Molecular Psychiatry 2020, Martyniuk et al., eLife 2022, Cazorla et al., Neuron 2014) |
| Data exclusions | Animals were excluded from fiber photometry experiments if no behaviorally-relevant or opto-induced signal could be detected, indicating failed surgery. Animals were excluded from local drug infusion experiments if the cannulas were clogged (drug reflux upon infusion). Animals were excluded if the implants or fell off, or if theydid not reach the right location, determined during post-hoc anatomy. Criterion were pre-established. |
| Replication | Results for anatomy showing that dSPN send collaterals to the GPe and SNr were shown twice with two methods (anterograde and retrograde tracing). Results from in vivo recordings of bridging collaterals was reproduced with two GCaMP sensors (GCaMP7s and Synaptophysin-GCaMP8s). Results from in vivo manipulation of bridging collaterals was reproduced with two methods: hM4D (chemogenetics) and eOPN3 (Gi/o coupled opsin). Results for in vivo recording of single units in GPe and SNr after chemogenetic dSPN manipulation were shown in 4 independent runs with different stimulation durations. Results from in vivo manipulation of dSPN and recording of Npas1 were reproduced 4 independent times with different stimulation protocols. Optogenetic stimulation of Npas1 and ChAT neurons and impact of motor function was evaluated in two independent tests (open field and rotarod). |
| Randomization | Mice were distributed randomly, matching litter/cage and sex. |
| Blinding | Experimenters were blinded for all experiments when this was experimentally possible. |

# Reporting for specific materials, systems and methods

We require information from authors about some types of materials, experimental systems and methods used in many studies. Here, indicate whether each material, system or method listed is relevant to your study. If you are not sure if a list item applies to your research, read the appropriate section before selecting a response.

## Materials & experimental systems

| n/a | Involved in the study | |
|---|---|---|
| | [x] | Antibodies |
| [x] | | Eukaryotic cell lines |
| [x] | | Palaeontology and archaeology |
| | [x] | Animals and other organisms |
| [x] | | Clinical data |
| [x] | | Dual use research of concern |

## Methods

| n/a | Involved in the study | |
|---|---|---|
| [x] | | ChIP-seq |
| [x] | | Flow cytometry |
| [x] | | MRI-based neuroimaging |

# Antibodies

Antibodies used

Chicken Anti-GFP (used 1:1000) Abcam Cat#Ab13970 (RRID AB_300798)
Rabbit Anti-GFP (used 1:2000) Molecular Probes Cat#A11122 (RRID AB_221569)
Rabbit Anti-DsRed (used 1:500) Clontech Cat#632496 (RRID AB_10013483)
Chicken Anti-mCherry (used 1:500) Abcam Cat#Ab205402 (RRID AB_2722769)
Mouse Anti-Synaptophysin (used 1:1000) Abcam Cat#Ab8049 (RRID AB_2198854)
Rabbit Anti-VGAT (1:500) Synaptic Systems Cat#131003 (RRID AB_887869)
Rabbit Anti-Cre (used 1:1000) (Kellendonk et al., 1999) In-house
Alexa 488 Anti-Chicken (used 1:1000) Thermofisher Cat#A11039 (RRID AB_142924)
Alexa 488 Anti-Rabbit (used 1:1000) Thermofisher Cat#A21206 (RRID AB_2535792)
Alexa 546 Anti-Rabbit (used 1:1000) Thermofisher Cat#A10040 (RRID: AB_2534016)
Alexa 546 Anti-Mouse (used 1:1000) Thermofisher Cat#A10036 (RRID AB_2534012)
Alexa 405 Anti-Rabbit (used 1:1000) Thermofisher Cat#A31556 (RRID AB_221605)
Biotinylated Anti-Rabbit (used 1:1000) Vector Laboratories BA-1000 (RRID AB_2313606)

Validation

We made the cre antibody and validated it in: Kellendonk, C. et al. Inducible site-specific recombination in the brain. J. Mol. Biol. 285, 175–182 (1999). GFP, DsRED, mCherry antibodies do not show signal in mice not receiving AAVs expressing GFP, DsRED, mCherry as they do not recognize endogenous proteins. They were validated for IHC, as state by the provider's website. Synaptophysin and VGAT antibodies were previously validated to recognize mouse endogenous proteins including for IHC see https://sysy.com/product/131003#list and https://www.abcam.com/synaptophysin-antibody-sy38-ab8049.html

# Animals and other research organisms

Policy information about studies involving animals; ARRIVE guidelines recommended for reporting animal research, and Sex and Gender in Research

Laboratory animals

Animals were housed under a 12-h light/12-h dark cycle in a temperature-controlled environment (22°C, humidity of 30–70%) with food and water available ad libitum, unless otherwise noted. Adult (>8 weeks old) male and female Drd1-GFP (X60Gsat/Mmmh; MMRRC), Drd1-cre (FK150Gsat/Mmcd; MMRRC), Npas1-cre (027718; Jackson; gift from S. Chan) and ChAT-cre (GM60; GENSAT) mice backcrossed onto C57BL/6 J background were used for experiments.

Wild animals

The study did not involve wild animals.

Reporting on sex

Findings apply to both sexes. Both males and females were used. Sex was not considered in study design. There are not enough animals per group to study interactions between sex and other variables. Sex data was not always saved and therefore data cannot be split per sex. Sex-based analyses was not done because on original pilot experiments, no sex-differences were detected.

Field-collected samples

The study did not involved samples collected in the field.

Ethics oversight

All animal procedures followed NIH guidelines and were approved by the New York State Psychiatric Institute or the National Institute on Drug Abuse and Johns Hopkins Medicine Animal Care and Use Committees.

Note that full information on the approval of the study protocol must also be provided in the manuscript.
